# Ovivorous Opportunities: Predation Events During Nest Guarding in a Freshwater Fish Species

**DOI:** 10.1002/ece3.70951

**Published:** 2025-03-12

**Authors:** Brett M. Studden, Timothy Fernandes, Bailey McMeans

**Affiliations:** ^1^ Department of Biology University of Toronto Mississauga Mississauga Ontario Canada; ^2^ Department of Ecology and Evolutionary Biology University of Toronto Toronto Ontario Canada

**Keywords:** egg predation, fish, freshwater, nesting, predator–prey interactions

## Abstract

The construction and defense of nests leading up to and following spawning is widespread across freshwater fishes. Despite the known role of nesting in sexual selection and the establishment of social hierarchy, how nesting and nest guarding behavior may shape predation risk for both offspring and nest guarding individuals remains relatively underexplored for many species. Here, we documented a novel interaction between a nest guarding fish species, the pumpkinseed sunfish ( *Lepomis gibbosus* ), and common snapping turtles ( *Chelydra serpentina* ) during the pumpkinseed nesting period. Field surveys were conducted over 3 days in an Ontario wetland to document nest densities, predator presence, and predation attempts by potential nest predators. A total of 118 pumpkinseed nests were observed, with all but five nests located in colonies. In nine instances, adult snapping turtles were recorded inspecting guarded pumpkinseed nests. On two occasions, snapping turtles were observed consuming nest contents, as indicated by a gulping motion while on the nest. Male pumpkinseed exhibited defensive behaviors, such as diversion and aggression. Following these interactions, we returned to quantify nest abandonment in three nests and observed that pumpkinseed had abandoned each nest within 48 h. While no observations of predation by snapping turtles on adult sunfish were made, other opportunistic predators made foraging attempts on guarding males. Though nest guarding can improve egg and offspring survival, our results document that predators are still capable of consuming nest contents even while guarded. Omnivorous foraging on fish eggs that are only briefly available to consumers but spatially aggregated in specific spawning habitats and nest colonies warrants future work evaluating the impact of nesting for: (1) adult and offspring survival across systems with divergent predator communities, and (2) the contribution of egg consumption to the seasonal energy budgets of egg predators.

## Graphical abstract

We investigate a rare interaction between pumpkinseed sunfish ( *Lepomis gibbosus* ) and opportunistic predators, namely common snapping turtles ( *Chelydra serpentina* ), during spring nesting. Turtles overcame active nest defenses by guarding males, highlighting the underappreciated role of nesting among freshwater fishes in generating aquatic food web interactions.

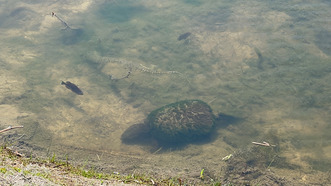

## 1 Introduction

Ovivory is common in the animal kingdom, with many species well adapted to identifying visual or olfactory cues generated by animal nests. For example, scent and soil disturbance allow foxes ( *Vulpes vulpes* ) to identify and predate turtle nests (Geller and Parker 2022; Spencer 2002). In fishes, egg predators commonly aggregate on spawning shoals or surrounding constructed nests to feed on any unguarded eggs (Mason and Evans 2011; Nester and Poe 1984). Parental care can improve offspring survival and occurs in approximately 60% of freshwater fish families, manifesting in various forms with differing degrees of care (Baylis 1981). Nesting involves modifying the surrounding environment to improve spawning and rearing habitat, and some fish species will also guard their nests to further reduce the risk of nest predation and offspring mortality (Steinhart et al. 2005). For example, when guarding smallmouth bass are removed from nests, nests experience markedly increased predation pressure and offspring mortality, wherein up to 60% of nests incurred predation within a minute of removal (Gravel and Cooke 2009). Although not commonly considered, nest creation and guarding could also function to aggregate both high‐quality eggs and guarding parental fish, making them more easily accessible to predators that can overcome parental defenses. For example, larger predators may target eggs and adults of smaller nest‐guarding species during the nesting period when vulnerability is potentially increased (Fernandes et al. 2024; Jones and Paszkowski 1997). Despite a rich body of literature investigating egg predation and nest guarding efficacy in larger species, such as smallmouth bass, few studies have documented instances of predation events on actively guarded fish nests in smaller‐bodied nest guarding species.

Nesting by freshwater fishes may function as a resource pulse event for many opportunist predators, where either high‐quality eggs or guarding parental fish become temporarily vulnerable to predation. Resource pulses in freshwater environments, from the decomposition of salmon carcasses following migratory runs (Wipfli et al. 1998) and the seasonal emergence of invertebrates providing energy and nutrients for both aquatic and terrestrial consumers (Baxter et al. 2005), can shape recruitment outcomes and stabilize competitive interactions (Bailey and Moore 2020; Ritchie and Colby 1988). The ability of predators to flexibly exploit ephemeral and abundant lower trophic level resources, from invertebrates to fish eggs, is an example of dynamic omnivory, which can also play a role in food web stability (Gutgesell et al. 2022; McCann et al. 1998). The predictability of spawning and nesting events may be a widespread mechanism that sets up foraging opportunities for predators. Because nest guarding can be energetically costly for parental individuals (Steinhart et al. 2005), it is also informative to consider the effectiveness of guarding for preventing nest predation, and whether guarding individuals themselves are susceptible to predation. Thus, the nesting period presents a promising opportunity for studying species interactions that have a wide range of potential consequences from parent and offspring survival to consumer–resource interactions and the broader community (Yang et al. 2008).

The present study examines predation attempts on pumpkinseed sunfish nests, hereafter pumpkinseed, during nest guarding. The colonial nature and synchrony of nesting pumpkinseed may give rise to potential opportunities for predators, seeking to exploit either nest contents or nest‐guarding individuals. We observed predation by opportunist predators across 3 days in an Ontario wetland and highlight the importance of documenting ecological interactions during fish nesting.

## 2 Methods

### 2.1 Site and Subjects

Big Creek National Wildlife Area (NWA) (Port Rowan, ON, Canada) is a 770‐ha wetland located on the perimeter of Lake Erie, supporting a diverse wildlife assemblage. The wetland is home to an array of predators, including birds, snakes, fishes, and turtles. Over 30 species of freshwater fish have been identified here, including pumpkinseed. The Hahn marsh habitat accommodates a natural population of pumpkinseed, which offer an excellent model for studying nest guarding as they form nesting colonies and perform complex courtship and guarding behaviors (Rios‐Cardenas and Webster 2005).

### 2.2 Data Collection

Between May 20, 2024 and May 23, 2024, pumpkinseeds were actively spawning, and males were nest guarding along the shore of the Big Creek Unit. Five 100 m transects were established along the Big Creek Trail loop. Estimates of pumpkinseed nest density were made by visual survey along each transect and extended two meters offshore. Nesting sites are characterized by a circular depression in the substrate with a male present, easily identified along the shoreline. We spent a total of 6 hours in search of and observing nest interactions over 3 days. When observed, snapping turtles were followed closely before, during, and after nest inspection and predation, noting the time spent on each nest. Nest inspection behavior was defined as a downward head posture with beaks near the nest substrate. To document the impact of nest interactions on potential nest abandonment, we returned to three nests within 48 h of each event. Any instances of predation on nesting males by snapping turtles or other predators were recorded.

## 3 Results

### 3.1 Nesting

Across the study area, we observed a total of 118 pumpkinseed nests with a transect density ranging from 0.065 to 0.155 nests/m^2^. Most nests were highly concentrated, with colonies separated by emergent vegetation near the shoreline. Five nests were identified to be solitary based on isolation from surrounding colonies. Several instances of active spawning were observed during surveys, but the majority of nests were occupied by a single guarding male on the nest.

### 3.2 Predation by Snapping Turtles

During shoreline surveys, we observed seven adult snapping turtles and one juvenile, five of which actively inspected pumpkinseed nests. Three turtles, not involved in predation attempts, were observed within approximately 3 m of pumpkinseed nesting colonies.

**FIGURE 1:**
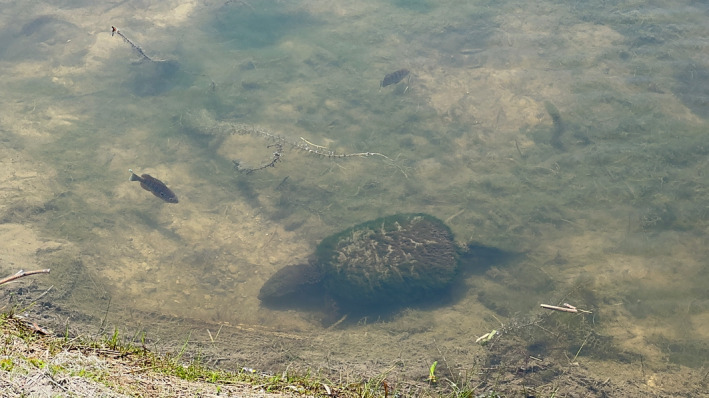
Photograph of an interaction between a male nest guarding pumpkinseed and a nest inspecting snapping turtle.

Nest inspection by adult snapping turtles ranged from 10 to 694 s (*n* = 9), with an average time of 259 s. Once at the nest, turtles positioned their head close to the substrate, appearing to search (Figure 1). During the two longest nest encounters (663 and 694 s), turtles actively consumed nest contents, as indicated by a guttural gulping motion. Following inspection and apparent egg predation, turtles exited the nesting area and moved to deeper water. In one case, an individual turtle interacted with a total of six nests, seemingly predating only one of the nests before moving into deeper water. On two occasions, snapping turtles approached and inspected nests with an actively mating pair of pumpkinseed, resulting in the dissolution of mating. Unfortunately, due to logistical constraints, of the nine recorded instances of nest inspection, we were only able to revisit three of the nests and each was abandoned by the male pumpkinseed.

### 3.3 Nest Guarding

During nest interactions between snapping turtles and pumpkinseed, pumpkinseed males generally stayed on or near the nest (Video 1). In many cases, this was the extent of the behavior exhibited by defending pumpkinseed males. However, some pumpkinseed demonstrated repeated diversion behavior occurring either during nest inspection or as the turtle was approaching. Only one instance of physical aggression was observed across all nine nest inspection observations. Here, the male repeatedly lunged at the head of the turtle until the turtle vacated the nest (Video 2).

**VIDEO 1:** Video of a nest interaction with the male pumpkinseed remaining in close proximity to the nest invading snapping turtle. The turtle appears to search the nest substrate with a downward facing head posture. Nearby males can be seen swimming around their respective nests. Video content can be viewed at https://onlinelibrary.wiley.com/doi/10.1002/ece3.70951

**VIDEO 2:** Video of a nest interaction where the guarding male exhibited aggression, lunging four times toward the head of the inspecting snapping turtle. Video content can be viewed at https://onlinelibrary.wiley.com/doi/10.1002/ece3.70951

Many instances of nest inspection by turtles were observed in close proximity to other nests with a guarding male pumpkinseed, but an altruistic behavioral response, such as mobbing was not elicited, despite being observed in other closely related species, such as bluegill ( *Lepomis macrochirus* ) (Dominey 1983). Instead, males in nearby nests began rapidly circling their own nests.

### 3.4 Other Interactions

During surveys, we observed several other opportunist predators among pumpkinseed nesting colonies. Leuciscids were observed within the perimeter of nests but were swiftly chased away by guarding males. Bowfin ( *Amia calva* ) were observed swimming among a small nesting colony. The bowfin did not demonstrate interest in nests; instead, the bowfin made several foraging attempts at nesting adult pumpkinseed (Video 3). No instances of egg predation attempts by pumpkinseed were made during surveys.

**VIDEO 3:** Video of a bowfin attempting to forage on an adult pumpkinseed in a nesting colony. The bowfin swims rapidly toward one of the nest guarding, but the nesting male evades the chase. Subsequently, the bowfin diverts its attention to another male in close proximity. Video content can be viewed at https://onlinelibrary.wiley.com/doi/10.1002/ece3.70951

## 4 Discussion

This study details a rarely documented predator–prey interaction between pumpkinseed and opportunistic snapping turtles during spring nesting. Although examples of nest predation by snapping turtles in the literature are uncommon, over 70% of observed adult turtles were found interacting with and, in some cases, foraging on pumpkinseed nests. Pumpkinseed exhibit an array of defensive behavioral strategies in response to nest predation by snapping turtles; however, they appear largely unsuccessful. Hence, the high concentration of nests and energy‐rich eggs give rise to foraging opportunities for snapping turtles even when guarded. Furthermore, guarding adults may be susceptible to predation by other fishes, as demonstrated by bowfin. Interestingly, no observations of predation by conspecifics were made despite the vast majority of males concentrated in nesting colonies. Colonial nesting may provide additional defensive opportunities and function to reduce predation pressure on any one individual, similar to fish schooling behavior (Magurran 1990). Although some debate exists regarding whether pumpkinseed are true colonial nesters (Gross 1982; Gross and MacMillan 1981), numerous cases of highly aggregated nesting support our observations of colonial nesting in the present study (Jordan et al. 2009; McPhail 2007; Scott and Crossman 1973; Ingram and Odum 1941; Krecker 1916). Future research could explore how local conditions or other factors influence the degree of colonial nesting in pumpkinseed sunfish.

Snapping turtles appear to exhibit egg predation more broadly prior to nesting in the early spring. For example, Moldowan et al. (2015) observed a female snapping turtle feeding on spotted salamander ( *Ambystoma maculatum* ) egg masses in Algonquin Provincial Park, Ontario. Remote underwater video surveys by Buse et al. (2017) then discovered four instances of inspection by snapping turtles on sunfish nests, with one confirmed instance of predation from 125 nest recordings. Based on these results, the authors concluded that nest interactions between pumpkinseed and snapping turtles were rare. Comparable to our study, nest inspection did not necessitate predation, which could relate to eggs not yet being released into the nest following construction. The unique accessibility for monitoring nearshore nesting sites in Big Creek NWA facilitated observations of such interactions. Observations made here thus suggest that nest predation behavior in snapping turtles may be more common than previously considered. The temporally restricted spawning window and relatively rapid maturation of offspring in nesting fishes like pumpkinseed may contribute to the current lack of consideration for ecological interactions during nest guarding.

In addition to the influence of egg predation on offspring mortality in fishes, energy and nutrients from egg predation may provide important resources for pre‐spawning snapping turtles. In Ontario, snapping turtles spawn in late spring, following a period of low availability of aquatic vegetation (Obbard and Brooks 1981). Other freshwater turtle species exhibit shifts toward carnivory during periods of low vegetative abundance before spawning (Petrov et al. 2024). Consumption of aggregated egg masses from nesting fishes may thus act as an ephemeral but high‐quality resource pulse rich in fat and protein (Kowalska‐Góralska et al. 2020). Our results identify a novel energy channel and highlight the potentially diverse dietary contributions to reproductive output in omnivorous turtle species, warranting further consideration.

Predator–prey interactions play a significant role in governing food web structure and dynamics (McCann et al. 1998). Though ephemeral and temporally dynamic interactions can be challenging to observe in the wild, such interactions can have drastic consequences for predator growth and behavior (Armstrong and Bond 2013). The extent to which nesting behavior and colonial spawning may function as important mediators of temporally structured predator–prey interactions remains largely unknown. As such, future work should consider the potential role of nesting in subsidizing the diet of opportunistic predators.

Despite this opportunity for predators, behavioral tactics employed by nest guarders are likely central to nesting and reproductive success (Gravel and Cooke 2009). Pumpkinseed are known to exhibit aggressive behaviors during nesting, ranging from opercular spreading, lunging, and nipping, making physical contact with an intruder (Stacey and Chiszar 1978). Although fine‐scale behaviors such as opercular spreading could not be observed, the majority of interactions did not involve physical aggression on the part of the guarding male. Appreciating the variation in nest guarding behaviors may be important for understanding the impact of novel or invasive predators on reproductive success and coping strategies of nesting freshwater fishes.

The present study outlines a unique interaction between nest guarding pumpkinseed and opportunistic predation by snapping turtles, providing newfound insights into the underappreciated role of nesting in generating predator–prey interactions in aquatic environments. The generality of egg predation in snapping turtles in other locations is currently unclear. However, nesting among freshwater fish species gives rise to several avenues for further study of the behaviors elicited by nest predation, their contribution to reproductive success against an array of predators, and the outcomes of nest predation for predator growth and food web dynamics. Future research should examine the costs of nest foraging opportunities for predators and seek to understand the extent to which nest predation may subsidize their diet, with careful consideration of its contribution to seasonal and reproductive energetics.

## Author Contributions

**Brett M. Studden:** conceptualization (lead), investigation (lead), methodology (lead), writing – original draft (lead), writing – review and editing (equal). **Timothy Fernandes:** conceptualization (supporting), writing – original draft (supporting), writing – review and editing (equal). **Bailey McMeans:** conceptualization (supporting), funding acquisition (lead), writing – original draft (supporting), writing – review and editing (equal).

## Conflicts of Interest

The authors declare no conflicts of interest.

## Data Availability

Data available through Dryad data repository, doi: https://doi.org/10.5061/dryad.7wm37pw30. Further inquiries may be directed to the corresponding author.
